# Timing of Umbilical Cord Blood Derived Mesenchymal Stem Cells Transplantation Determines Therapeutic Efficacy in the Neonatal Hyperoxic Lung Injury

**DOI:** 10.1371/journal.pone.0052419

**Published:** 2013-01-21

**Authors:** Yun Sil Chang, Soo Jin Choi, So Yoon Ahn, Dong Kyung Sung, Se In Sung, Hye Soo Yoo, Won Il Oh, Won Soon Park

**Affiliations:** 1 Department of Pediatrics, Samsung Medical Center, Sungkyunkwan University School of Medicine, Seoul, Korea; 2 Samsung Biomedical Research Institute, Sungkyunkwan University School of Medicine, Seoul, Korea; 3 Biomedical Research Institute, MEDIPOST Co., Ltd., Seoul, Korea; University of Sao Paulo – USP, Brazil

## Abstract

Intratracheal transplantation of human umbilical cord blood (UCB)-derived mesenchymal stem cells (MSCs) attenuates the hyperoxia-induced neonatal lung injury. The aim of this study was to optimize the timing of MSCs transplantation. Newborn Sprague-Dawley rats were randomly exposed to hyperoxia (90% for 2 weeks and 60% for 1 week) or normoxia after birth for 21 days. Human UCB-derived MSCs (5×10^5^ cells) were delivered intratracheally early at postnatal day (P) 3 (HT3), late at P10 (HT10) or combined early+late at P3+10 (HT3+10). Hyperoxia-induced increase in mortality, TUNEL positive cells, ED1 positive alveolar macrophages, myeloperoxidase activity and collagen levels, retarded growth and reduced alveolarization as evidenced by increased mean linear intercept and mean alveolar volume were significantly better attenuated in both HT3 and HT3+10 than in HT10. Hyperoxia-induced up-regulation of both cytosolic and membrane p47*^phox^* indicative of oxidative stress, and increased inflammatory markers such as tumor necrosis factor-α, interleukin (IL) -1α, IL-1β, IL-6, and transforming growth factor-β measured by ELISA, and tissue inhibitor of metalloproteinase-1, CXCL7, RANTES, L-selectin and soluble intercellular adhesion molecule-1 measured by protein array were consistently more attenuated in both HT3 and HT3+10 than in HT10. Hyperoxia-induced decrease in hepatocyte growth factor and vascular endothelial growth factor was significantly up-regulated in both HT3 and HT3+10, but not in HT10. In summary, intratracheal transplantation of human UCB derived MSCs time-dependently attenuated hyperoxia-induced lung injury in neonatal rats, showing significant protection only in the early but not in the late phase of inflammation. There were no synergies with combined early+late MSCs transplantation.

## Introduction

Recent improvements in neonatal intensive care medicine have resulted in marked improvements in the survival of the premature infants [1]. However, bronchopulmonary dysplasia (BPD), a chronic lung disease that follows ventilator and oxygen therapy in the premature infants, still remains a major cause of mortality and morbidity with few effective treatments [2], [3].

Although the pathogenesis of BPD has not been clearly elucidate yet, oxidative stress and the ensuing inflammation mediated by neutrophils [4] and pro-inflammatory cytokines [5] is believed to play a seminal role in the lung injury process leading to the development of BPD [6]. Recently, we have shown that local intratracheal but not systemic intraperitoneal xenotransplantation of human umbilical cord blood (UCB)-derived mesenchymal stem cells (MSCs) attenuates hyperoxia induced lung injuries such as impaired alveolarization, increased apoptosis and fibrosis in the immunocompetent neonatal rats [7]. Furthermore, these protective effects of stem cell transplantation were dose dependent [8]. Overall, these findings suggest that human UCB derived MSCs transplantation could be a novel therapeutic modality for BPD. However, while the administration of human UCB-derived MSCs at postnatal day (P) 5 was effective in our previous studies [7], [8], the optimal timing for their administration has not been determined yet.

Previously, we have shown that the protective effects of human UCB-derived MSCs transplantation are primarily mediated by their anti-inflammatory effects rather than by their regenerative capabilities [7], [8]. These findings suggest that the therapeutic time window of stem cell transplantation could be narrow, i.e., only during the early but not the late phase of inflammatory responses. In the present study, we thus tried to determine the optimal timing at which intratracheally delivered human UCB-derived MSCs could attenuate the hyperoxia-induced lung injuries in the newborn rat pups. We firstly conducted time course experiments of inflammatory responses by measuring inflammatory cytokines such as tumor necrosis factor (TNF)-α, interleukin (IL)-1α, 1β and 6 levels at P 0, 3, 5, 7, 10 and 14 in the hyperoxia-induced neonatal lung tissue. After then, we tried to determine the optimal timing by comparing the therapeutic efficacy of early (P3) versus late (P10) intratracheal administration of human UCB derived MSCs in attenuating the hyperoxia-induced lung injuries in the newborn rat pups. We also tried to determine whether combined early (P3)+late (P10) stem cell transplantation has any synergistic effects.

## Materials and Methods

### Cell Preparation

This study was approved by Institutional Review Board of Samsung Medical Center and by Medipost, Co., Ltd, Seoul, Korea. As previously reported, UCB was collected from umbilical veins after neonatal delivery with informed consent from pregnant mothers, and MSCs were isolated and cultivated from human UCB [9], [10]. The cells expressed CD105 (99.6%) and CD73 (96.3%), but not CD34 (0.1%), CD45 (0.2%) and CD14 (0.1%) [7]. They were positive for HLA-AB (96.8%), but generally not for HLA-DR (0.1%). The cells also expressed pluripotency markers such as octamer-binding transcription factor 4 (Oct 4; 30.5%) [11] and stage-specific embryonic antigen 4 (SSEA-4; 67.7%) [12]. Human UCB-derived MSCs differentiated into various cell types such as respiratory epithelium, osteoblasts, chondrocytes and adipocytes with specific *in vitro* induction stimuli [7], [10], [12], [13]. We confirmed the differentiation potential and karyotypic stability of the human UCB-derived MSCs up to the 11^th^ passage.

### Animal model

The experimental protocols described herein were reviewed and approved by the Animal Care and Use Committee of Samsung Biomedical Research Institute, Seoul, Korea. This study was also performed in accordance with the institutional and National Institutes of Health guidelines for laboratory animal care. Timed pregnant Sprague-Dawley rats (Orient Co., Seoul, Korea) were housed in individual cages with free access to water and laboratory chow. The rat pups were delivered spontaneously and reared with their dams. The experiment began within 10 h after birth, and continued through P21. Rat pups were randomly divided into four experimental groups; normoxia control group (NC), hyperoxia control group (HC), hyperoxia with early at P3 (HT3), late at P10 (HT10), or combined early+late at P3+10 (HT3+10) human UCB-derived MSCs transplantation group. Rat pups of NC were kept with a nursing mother rat in the standard cage at room air throughout the experiment. Rat pups of hyperoxia groups were maintained with a nursing mother in the standard cage within a 50 liter Plexiglas chambers in which the hyperoxia (oxygen concentration of 90%) was maintained until P14, and after then oxygen concentration was reduced to 60% until P21. Humidity and environmental temperature were maintained at 50% and 24°C, respectively. Nursing mother rats were rotated daily between litters in the normoxia and hyperxoxia groups to avoid oxygen toxicity. Survival and body weight of rat pups in each group were checked daily throughout the experiment. The rat pups of NC and HC were sacrificed at P 1, 3, 5, 7, 10 and 14 for time course experiments and at P21 for group comparison under deep pentobarbital anesthesia (60 mg/kg, intraperitoneal), and the whole lung tissue was obtained for morphometric and biochemical analyses. Six to eight animals were used in each subgroup of analysis.

### Transplantation of human UCB-derived MSCs

The human UCB-derived MSCs from the 5^th^ passage from a single donor were labeled using a PKH26GL Red Fluorescent Cell Membrane Labeling Kit (Sigma-Aldrich, St. Louis, MO, USA) for transplantation according to the manufacturer's protocol in the present study, as previously reported [7], [8]. For donor cell transplantation, 5×10^5^ cells in 0.05 ml phosphate buffered saline (PBS, pH 7.4) were administered intratracheally at P 3, P 10 or P 3+10. For NC and HC, equal volume of PBS was given intratracheally at P3 and P10. For intratracheal transplantation, the rats were anesthetized with an intraperitoneal injection of ketamine and xylazine mixture (45 mg/kg and 8 mg/kg, respectively), and restricted on a board at a fixed angle. MSCs were administered into the trachea through a 30-gauge needle syringe. After the procedure, the animals were allowed to recover from anesthesia, and were returned to their dams. There was no mortality associated with the transplantation procedure.

### Tissue preparation

The lungs were resected after transcardiac perfusion with ice-cold phosphate buffered saline (PBS), snap-frozen in liquid nitrogen, and stored at −80°C for later biochemical analyses.

For morphometric analyses, lungs were fixed in situ by tracheal instillation of 10% buffered formalin at a constant inflation pressure of 20 cm H_2_O, and then fixed overnight at room temperature in the same fixative. The fixed right lungs were embedded in paraffin, and the left lungs were embedded in an optimal cutting temperature (OCT) compound (SAKURA 4583, Sakura, Torrance, CA, USA). Blocks of the OCT compound were sectioned at 10 µm on a cryostat (Shandon Cryotome, Thermo Electron Co., Waltham, MA, USA) and stored in a deep freezer until analyzed by immunohistochemistry. Four-micrometer-thick sections were cut from the paraffin blocks, and stained with hematoxylin and eosin. Images of each section were captured with a magnifier digital camera through an Olympus BX40 microscope (Olympus optical Co. Ltd., Tokyo, Japan), and were saved as JPEG files.

### Morphometry

The level of alveolarization was determined by measuring the MLI and mean alveolar volume. The mean inter-alveolar distance was measured as MLI, by dividing the total length of the lines drawn across the lung section by the number of intercepts encountered, as described by Thurlbeck [14]. The mean alveolar volume was calculated using the method reported by Snyder et al. [15], [16]. Briefly, a grid containing equally spaced crosses was placed on a uniformly enlarged photomicrograph of each lung field. The diameters (ℓ) of the alveoli containing a cross were measured along the horizontal axis of the cross. The cube of the alveolar diameter times π and divided by 3 (ℓ^3^π/3) was used to estimate the mean alveolar volume. A minimum of two sections per rat and six fields per each section were examined randomly for each analysis.

### TUNEL assay

The immunofluorescent TUNEL staining with an in situ cell death detection kit (S7110 ApopTag, Chemicon, Temecula, CA, USA) was done to measure the extent of apoptosis in the lung. Paraffin section slides were deparaffinized, rehydrated, and digested with Proteinase K (20 µg/ml in PBS) (Sigma Co., St. Louis, MO, USA) at room temperature for 15 minutes and then washed in PBS for 10 minutes. Sections were then incubated with equilibration buffer for 1 minute and immediately incubated with working strength TdT enzyme in a humidified chamber at 37°C for 1 hour. Each section was immersed in a stop/wash buffer and gently rinsed with PBS. Fluorescein isothiocyanate (FITC)-labeled anti-digoxigenin conjugate was applied to the sections which were then incubated at room temperature for 30 minutes in the dark. Nuclear counterstaining was performed with propidium iodide (0.5 µg/ml, Sigma Co., St. Louis, MO, USA). Slides were washed again in PBS, mounted with Vectasheild mounting solution (Vector laboratories, Burlingame, CA, USA), and visualized with a fluorescent microscope (Nikon E600 fluorescence microscope, Tokyo, Japan) using an excitation wavelength of 460–490 nM. Ten non-overlapping fields with a magnifying power of ×200 were examined to count TUNEL positive cells.

### Quantification of the PKH26 positive cells

Ten-µm-thick cryosections were mounted with a Vector shield mounting solution containing DAPI (H-1200, Vector, Burlingame, CA, USA). The cell counts for the transplanted or donor-derived cells were measured using PKH26 red fluorescence, as described above after combining the ×20 objective images of the DAPI-stained nuclei signals. Five fields per section were selected randomly, focused, and counted with the naked eye under a fluorescence microscope (Nikon E600, Nikon, Tokyo, Japan) using a filter to detect the PKH26 red fluorescence. The PKH26 red fluorescence was counted manually and averaged per high power field (HPF) in a single animal. Two random sections per animal were evaluated in a blinded manner.

### Western blot for p47*^phox^*

The upregulation of p47*^phox^*, a subunit of NADPH oxidase, both in the cytoplasmic and plasma membrane portion serves as an indicator of NADPH oxidase activation that is responsible for generating reactive oxygen species [17], [18]. Tissue slides were incubated for 40 min in PBS containing 0.1% Triton X-100. After blocking with 0.5% BSA, slides were incubated overnight at 4°C with anti-p47*^phox^* antibody (1∶200) and then exposed for 2 hr at room temperature to FITC-conjugated goat anti mouse immunoglobulin-G (1∶200; BD Biosciences, USA). Vectasheld mount medium with DAPI (Vector Laboratories) was used to preserve. Confocal microscopic examination was carried out at 400× magnification using Bio-Rad Radiance 2100 (Bio-Rad Laboratories Inc. Hercules, CA, USA) with krypton/argon laser, and images were achieved using the Laser shop 2000 software (Bio-Rad Laboratories, Inc.). For this purpose, tissue sample from each animal were separated into membrane and cytosolic components for western blot examination. Tissues were homogenized in ice-cold hypertonic solution and centrifuged at 600 g for 10 minutes. The supernatant was ultra-centrifuged at 100,000 g for 1.5 hours. The supernatant contained the cytosolic fraction and the membrane-particulate pellet was resuspended in hypotonic solution containing 1% Triton X-100. Samples were analyzed by western blotting using antibodies against the NADPH oxidase cytosolic subunit, p47*^phox^* (1∶500, BD Biosciences, San Diego, CA, USA). The bands were recognized by horseradish peroxidase-conjugated anti-mouse secondary antibody (1∶1,000, DAKO, Glostrup, Denmark), and then western blots were developed with enhanced chemiluminescence detection reagents (Amersham Pharmacia, Uppsala, Sweden), and exposed to X-ray film (Fuji Photo Film, Tokyo, Japan). The blots were re-probed with antibodies against GAPDH (1∶1,000, Santa Cruz Biotechnology Inc., Santa Cruz, CA, USA). To determine the relative degree of membrane purification, the membrane fraction was subjected to immunoblotting for calnexin (1∶500 Santa Cruz Biotechnology Inc), a membrane marker.

### Protein macroarray

Each lung lysate was analyzed using a rat cytokine array kit (Proteome Profiler™; R&D Systems, Minneapolis, MN, USA). A total of 250 µg of lysate was incubated in the nitrocellulose membrane array overnight at 4°C. After washing away the unbound protein, the array was incubated with a cocktail of phospho-site-specific biotinylated antibodies for 2 h at room temperature, followed by streptavidin–HRP for 30 min. Signals were visualized with chemiluminescent reagents (Amersham Biosciences, Pittsburgh, PA, USA), and recorded on X-ray film. The arrays were scanned, and optical densities were measured using Image J software (NIH) and compared among the experimental groups. The protein macroarray analysis included inflammatory cytokines of interest, including tissue inhibitor of metalloproteinase (TIMP)-1, Chemokine (C-X-C motif) ligand 7 (CXCL7), regulated upon activation normal T-expressed and presumably secreted (RANTES), L-selectin and the soluble form of intercellular adhesion molecule (sICAM)-1.

### Myeloperoxidase activity

The activity of MPO, an indicator of neutrophil accumulation, was determined by modification of the method by Gray et al. [19]. The lung tissues were homogenized in a phosphate buffer (pH 7.4) and centrifuged at 30,000 g for 30 min. The pellet was resuspended in another phosphate buffer (50 mM, pH 6.0) containing 0.5% hexadecyltrimethyl ammonium bromide. MPO activity in the resuspended pellet was assayed by measuring absorbance changes spectrophotometrically at 460 nm, using 0.167 mg/mL of O-dianisidine ihydrochloride and 0.0005% hydrogen peroxide. One unit of MPO activity was defined as the quantity of enzyme degrading 1 µM of peroxide/min.

### Hepatocyte growth factor (HGF)

Total RNA in the sample was extracted using RNA Trizol according to the manufacturer's protocol (Invitrogen Corporation, Carlsbad, CA, USA). Total RNA concentration was measured by spectrophotometry (Nanodrop Wilmington, DE, USA) at 260 nm. One microgram of RNA was used to produce cDNA with a Protoscript® II RT-PCR kit (New England Biolabs, Ipswich, MA, USA). PCR primers for rat hepatocyte growth factor and rat glyceraldehyde-3-phosphate dehydrogenase (GAPDH) were designed with Primer3 (Whitehead Institute, Cambridge, MA, USA) and synthesized by Bioneer Inc. (Bioneer, Daejeon, Korea). The sequence of primers used was as follows: rat HGF (sense-accctggtgtttcacaagca- antisense-aggggtgtcagggtcaagag-), rat GAPDH (sense- ggccaaaagggtcatcatct-, antisense-gtgatggcatggactgtggt-). PCR products were run on a 1.2% agarose gel electrophoresis, visualized by ethidium bromide and scanned by a Gel Doc 2000 analyzer (Bio-Rad, Hercules, CA, USA). The expression levels for each gene were semi-quantified by densitometric analysis using software (Quantity One, Bio-Rad, Hercules, CA, USA). Relative expression levels were estimated by the density ratio of rat GAPDH to rat HGF.

### Statistical Analysis

The data are expressed as the mean ± SEM. Survival rates were compared using the Kaplan-Meier analysis followed by a log rank test. For continuous variables with a normal distribution, the groups were compared using a *t*-test with a Bonferroni correction. Continuous variables that were not normally distributed were analyzed using the Wilcoxon rank test with a Bonferroni correction. All data were analyzed using Stata software (ver. 11.0, StataCorp LP, College Station, TX, USA). Values of *p*<0.05 were considered statistically significant.

## Results

### Temporal profile of inflammatory responses

**Figure 1:**
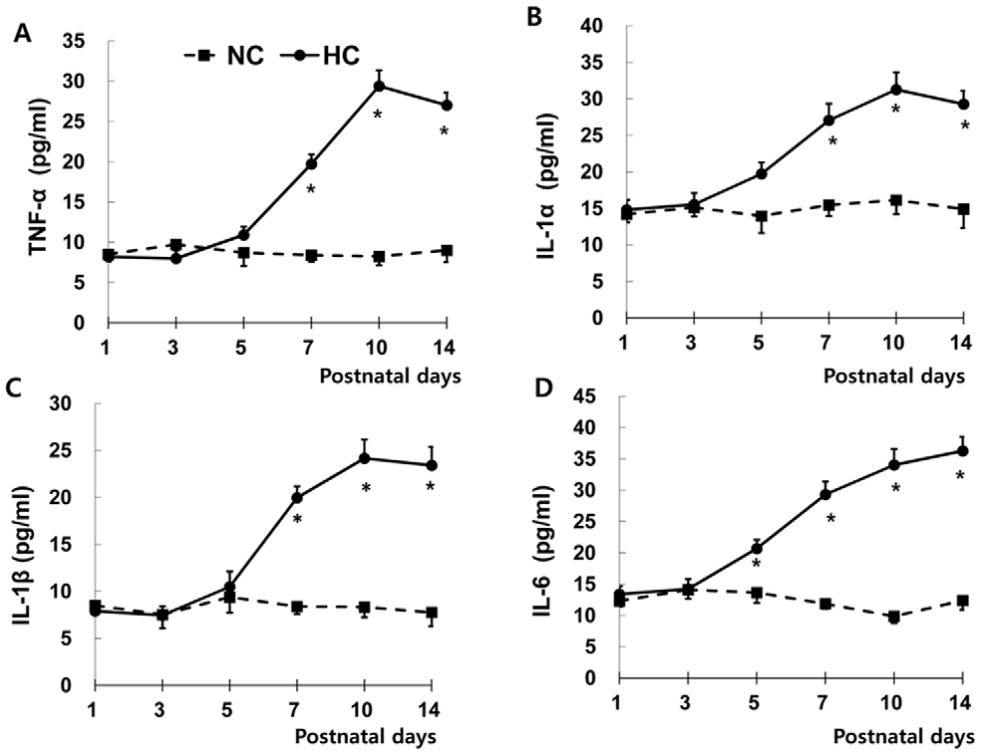
Temporal profiles of inflammatory cytokines. Tumor necrosis factor (TNF)-α, interleukin (IL)-1α, IL-1β, and IL-6 levels measured with ELISA at P 1, 3, 5, 7, 10 and 14 in the rat lung tissue. NC, Normoxia control group; HC, hyperoxia control group. Data; mean±SEM. ^*^ *P*<0.05 compared to NC.

In time course experiments of inflammatory responses by measuring TNF-α, IL-1α, IL-1β and IL-6 levels with ELISA at P 1, 3, 5, 7, 10 and 14 in the lung tissue, IL-6 levels after P5 and TNF-α, IL-1α and IL-1β levels after P7 in HC became significantly increased compared to NC up to P 14 (Fig. 1).

### Survival rate and body weight gain

**Figure 2:**
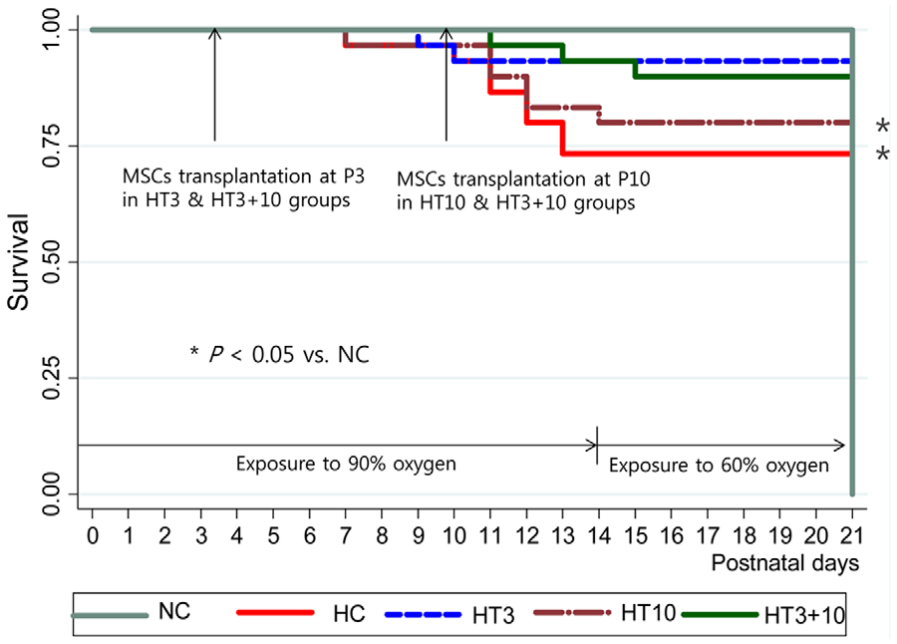
Survival curve. Kaplan-Meier survival curve up to P 21 showing decreased survival. HC compared to NC, and improved survival in both HT3 and HT3+10, but not in HT10. NC, Normoxia control group; HC, hyperoxia control group; HT3, hyperoxia with stem cell transplantation group at P3; HT10, hyperoxia with stem cell treatment group at P10; HT3+10, hyperoxia with stem cell treatment group at P3 and P10. ^*^ *P*<0.05 compared to NC.

Exposure to oxygen (HC) significantly reduced the survival rate to 70.8% (*P*<0.05 vs. NC) at the end of experiment (P21) compared to the 100% survival rate of NC. On the contrary, survival rates of HT3 (91.7%, *P*>0.05 vs. NC) and HT3+10 (87.5%, *P*>0.05 vs. NC) were not different when it compared to NC. However, survival rate of HT10 (75.0%, *P*<0.05 vs. NC) was significantly lower than that of NC (Fig. 2).

Although birth weight was not significantly different between the five experimental groups (7.0±0.13 g, 7.1±0.04 g, 7.1±0.04 g, and 7.1±0.03 in NC, HC, HT3, HT10 and HT3+10, respectively), body weight at P21 in HC (34.3±4.6 g, *P*<0.01) was significantly lower compared to NC (41.7±2.3 g), and this retarded body weight gain observed in HC was significantly improved in HT3 (39.5±4.7 g, *P*<0.01 vs. HC) and HT3+10 (38.8±4.4 g, *P*<0.01 vs. HC), but not in HT10 (35.7±4.2 g, *P*>0.05 vs. HC, *P*<0.05 vs. HT3, *P*<0.05 vs. HT3+10 ).

### Lung histopathology

**Figure 3:**
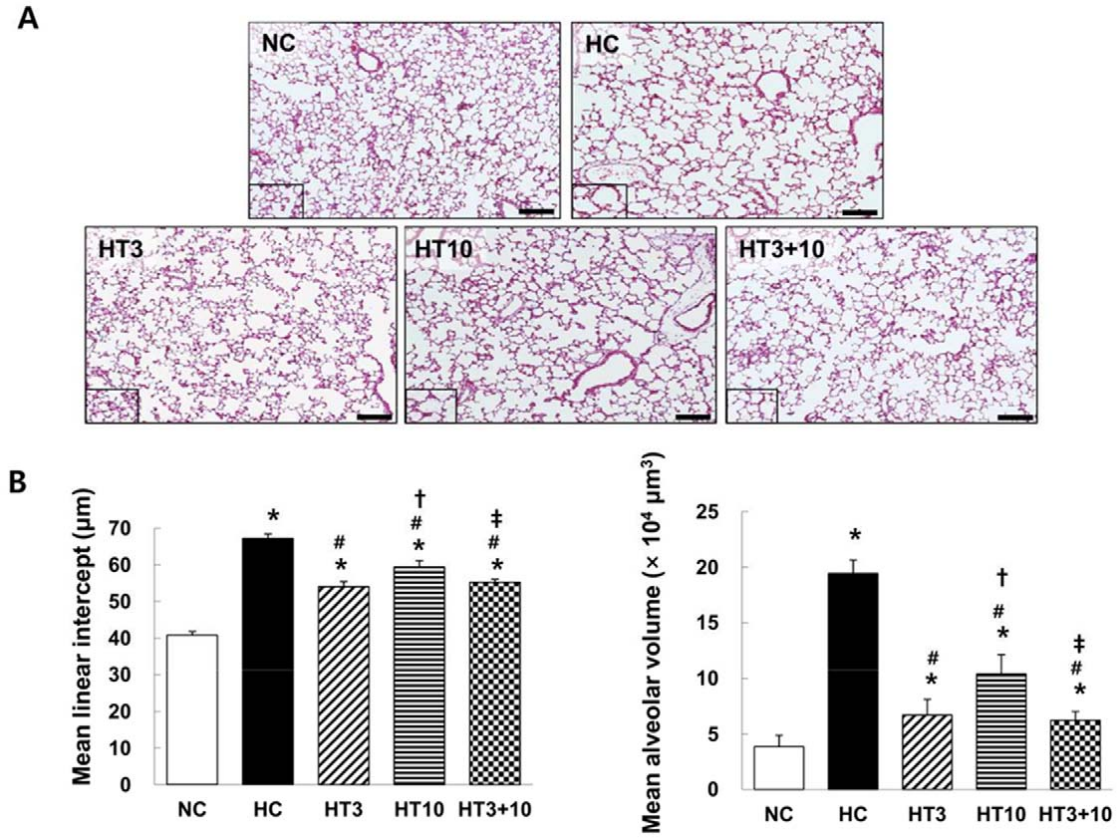
Histology and morphometric analysis of the surviving P21 rat lung. (A): Representative optical microscopy photomicrographs of the lungs stained with hematoxylin and eosin (scale bar = 100 µm). (B): Degree of alveolarization measured by the mean linear intercept (*left*) and mean alveolar volume (*right*). NC, Normoxia control group; HC, hyperoxia control group; HT3, hyperoxia with stem cell transplantation group at P3; HT10, hyperoxia with stem cell treatment group at P10; HT3+10, hyperoxia with stem cell treatment group at P3 and P10. Data; mean±SEM. ^*^ *P*<0.05 compared to NC, ^#^ *P*<0.05 compared to HC,^†^ *P*<0.05 compared to HT3, ^‡^ *P*<0.05 compared to HT10.

Fig. 3A presents typical photomicrographs showing the histopathological differences observed by optical microscopy in each experimental group at P 21. While uniform and small alveoli were observed in NC, impaired alveolar growth, as evidenced by fewer and larger alveoli, focal airspace enlargement and heterogeneous alveolar size were observed in HC compared to NC. After MSCs transplantation, the hyperoxia-induced impairments in alveolar growth and morphological changes were attenuated, particularly in both HT3 and HT3+10 compared to HT10.

In morphometric analyses, the MLI and mean alveolar volume, indicating the size and volume of the alveoli respectively, were significantly higher in HC (67.2±1.2 µm in MLI, *P*<0.001 vs. NC; 19.4±1.2×10^4^ µm^3^ in mean alveolar volume, *P*<0.001 vs. NC) than in NC (40.8±1.0 µm in MLI and 3.1±0.04×10^4^ µm^3^ in mean alveolar volume). (Fig. 3B). After MSCs transplantation, these hyperoxia-induced morphometric abnormalities were better attenuated in HT3 (54.0±1.4 µm in MLI, *P*<0.001 vs. HC; 6.7±0.9×10^4^ µm^3^ in mean alveolar volume *P*<0.001 vs. HC) and HT3+10 (55.3±0.8 µm vs. HC, *P*<0.001; 6.2±0.5×10^4^ µm^3^ in mean alveolar volume *P*<0.001 vs. HC) compared to those in HT10 (59.4±1.7 µm in MLI, *P*<0.001 vs. HC, *P*<0.01 vs. HT3, , *P*<0.05 vs. HT3+10 ; 10.4±1.0×10^4^ µm^3^ in mean alveolar volume *P*<0.001 vs. HC, *P*<0.01 vs. HT3, *P*<0.01 vs. HT3+10) (Fig. 3B).

**Figure 4:**
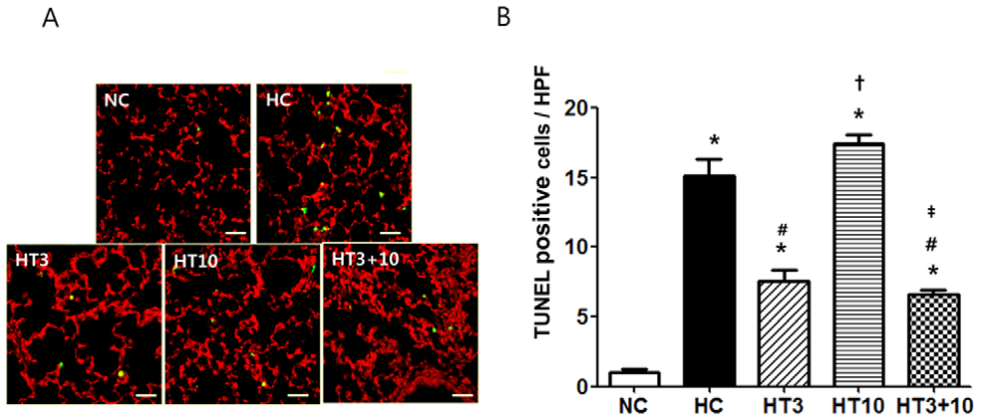
TUNEL positive cells in the distal lungs of the P 21 rat pups. (A): TUNEL positive cells were labeled with FITC (*green*) and the cell nuclei were labeled with propidium iodide (*red*) (Scale bar; 25 µm). (B): Number of observed TUNEL positive cells per high power field. NC, Normoxia control group; HC, hyperoxia control group; HT3, hyperoxia with stem cell transplantation group at P3; HT10, hyperoxia with stem cell treatment group at P10; HT3+10, hyperoxia with stem cell treatment group at P3 and P10. Data; mean±SEM. ^*^ *P*<0.05 compared to NC, ^#^ *P*<0.05 compared to HC,^†^ *P*<0.05 compared to HT3, ^‡^ *P*<0.05 compared to HT10.

The number of TUNEL positive cells in the lung of P21 rats per high power field was significantly increased in HC (15.2±1.1, *P*<0.001) compared to NC (1.1±0.2). This hyperoxia-induced increase in the number of TUNEL positive cells was significantly attenuated in both HT3 (7.6±0.8, *P*<0.001 vs. HC) and HT3+10 (6.6±0.3, *P*<0.001 vs. HC), but not in HT10 (17.4±0.6, *P*>0.05 vs. HC, *P*<0.001 vs. HT3, *P*<0.001 vs. HT3+10) (Fig. 4).

**Figure 5:**
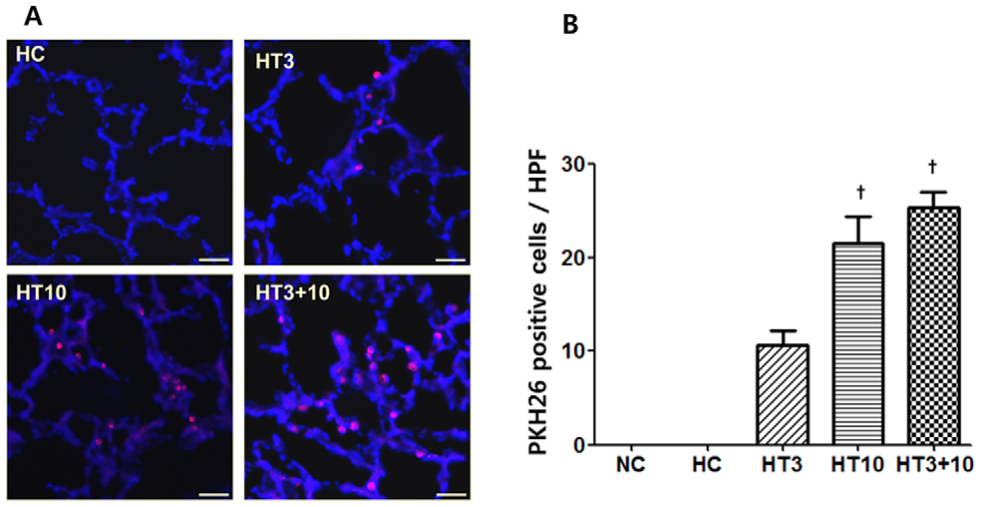
Donor cell localization in the lung of the P 21 rats. (A): Fluorescent microscopic observation of the PKH26 labeled human UCB-derived MSCs (donor cells, *red*) localized in the lungs of the P 21 newborn rats and the nuclei labeled with DAPI (*blue*) (Scale bar; 25 µm). (B): Number of PKH26 positive cells in the lung per high power field. NC, Normoxia control group; HC, hyperoxia control group; HT3, hyperoxia with stem cell transplantation group at P3; HT10, hyperoxia with stem cell treatment group at P10; HT3+10, hyperoxia with stem cell treatment group at P3 and P10. Data; mean±SEM. ^†^ *P*<0.05 compared to HT3.

The deposition of PKH26 red fluorescence positive donor cells was observed only in the MSCs transplantation groups, but not in NC and HC (Fig. 5A). The number of donor cells identified per lung field was significantly larger in HT10 (21.5±2.9, *P*<0.001 vs. HT3) and HT3+10 (25.4±1.7, *P*<0.001 vs. HT3) than in HT3 (10.6±1.6). However, there were no significant differences in the donor cells between HT10 and HT3+10 (Fig. 5B).

### Cytosolic and membrane expressions of p47*^phox^*

**Figure 6:**
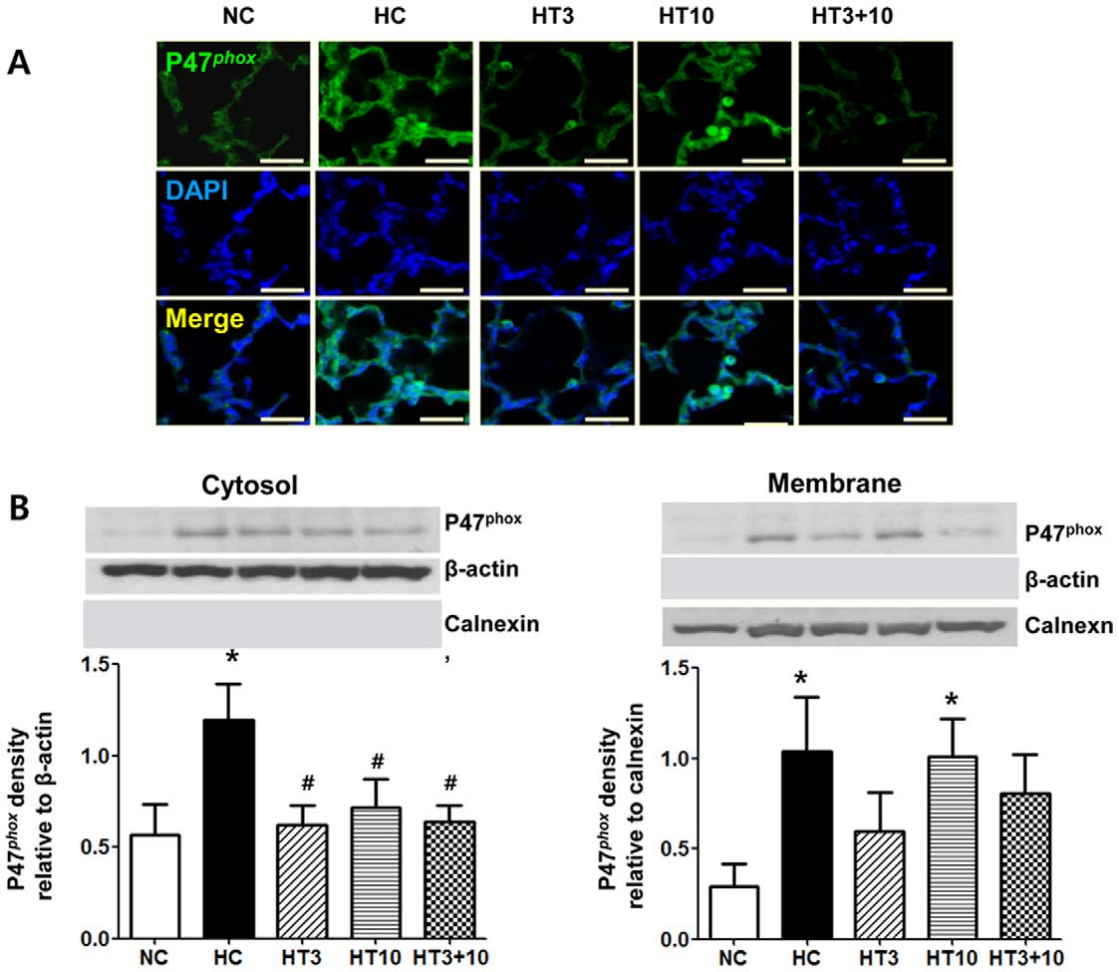
p47*^phox^*, cytosolic subunit of nicotinamide adenine dinucleotide phosphate oxidase in the P21 rat lung tissue. (A): Fluorescent microscopic observation of p47*^phox^* (*green*) localized in the lungs of the P21 rats and the nuclei labeled with DAPI (*blue*) (Scale bar; 25 µm). (B): Representative western blots (*top*) and densitometric histograms (*bottom*) in the cytosol (*left*) and membrane (*right*) fractions of P21 rat lung homogenates. NC, Normoxia control group; HC, hyperoxia control group; HT3, hyperoxia with stem cell transplantation group at P3; HT10, hyperoxia with stem cell treatment group at P10; HT3+10, hyperoxia with stem cell treatment group at P3 and P10. Data; mean±SEM. ^*^ *P*<0.05 compared to NC, ^#^ *P*<0.05 compared to HC.

Since NADPH oxidase produces oxygen free radicals in both phagocytic [20] and nonphagocytic cells [21], [22], hyperoxia-induced production of ROS was evaluated by NADPH oxidase activation, as evidenced by increased cytosolic and membrane expression of a cytosolic subunit of NADPH oxidase p47*^phox^*. In fluorescent microscopy, increased p47*^phox^* was observed in HC compared to NC, indicating the activation of NADPH oxidase. After MSCs transplantation, the hyperoxia-induced increase in p47*^phox^* was attenuated, particularly in both HT3 and HT3+10 compared to HT10 (Fig. 6A). In western blot analyses, significantly higher levels of p47*^phox^* were observed in HC both in the cytosolic (*P*<0.01) and membrane (*P*<0.05) fractions than in NC. While the hyperoxia-induced increase in the cytosolic expression of p47*^phox^* was significantly attenuated in the MSCs transplantation groups (*P*<0.05 vs. HC) the increase in the membrane fraction was attenuated in both HT3 (*P*>0.05 vs. NC) and HT3+10 (*P*>0.05 vs. NC), but not in HT10 (*P*<0.05 vs. NC).

### ELISA and Protein array of cytokines

**Figure 7:**
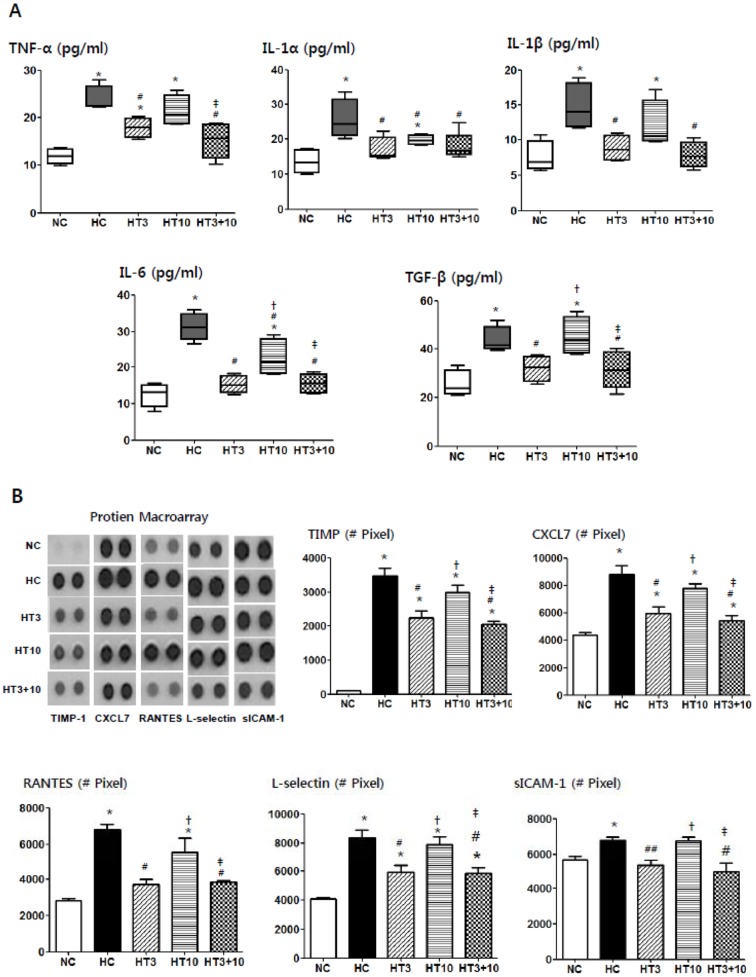
Histograms of inflammatory cytokines and chemokines in the hyperoxic lung injury after MSCs transplantation. TNF-α, IL-1α, IL-1β, IL-6, and TGF-β levels measured by ELISA (A) and TIMP-1, CXCL7, RANTES, L-selectin and sICAM-1 levels measured by protein array at P21 in the rat lung tissue (B,C). NC, Normoxia control group; HC, hyperoxia control group; HT3, hyperoxia with stem cell transplantation group at P3; HT10, hyperoxia with stem cell treatment group at P10; HT3+10, hyperoxia with stem cell treatment group at P3 and P10. Data; mean±SEM. ^*^ *P*<0.05 compared to NC, ^#^ *P*<0.05 compared to HC,^†^ *P*<0.05 compared to HT3, ^‡^ *P*<0.05 compared to HT10.

In HC, significantly increased levels of TNF-α (*P*<0.001 vs. NC), IL-1α (*P*<0.001 vs. NC), IL-1β (*P*<0.01 vs. NC), IL-6 (*P*<0.001 vs. NC) and TGF-β (*P*<0.01 vs. NC) measured by ELISA and TIMP-1 (*P*<0.001 vs. NC), CXCL7 (*P*<0.001 vs. NC), RANTES (*P*<0.001 vs. NC), L-selectin (*P*<0.001 vs. NC) and sICAM-1 (*P*<0.05 vs. NC) measured by protein array were observed compared to NC (Fig. 7). The hyperoxia-induced increase in these cytokine levels was significantly attenuated in both HT3 and HT3+10, but not in HT10, and the attenuation of IL-1α and IL-6 was more profound in HT3 (IL-1α, *P*>0.05 vs. NC, *P*<0.01 vs. HC; IL-6, *P*>0.05 vs. NC, *P*<0.001 vs. HC) and HT3+10 (IL-1α, *P*>0.05 vs. NC, *P*<0.01 vs. HC; IL-6, *P*>0.05 vs. NC, *P*<0.001 vs. HC) than in HT10 (IL-1α, *P*<0.05 vs. NC, *P*<0.05 vs. HC; IL-6, *P*<0.01 vs. NC, *P*<0.01 vs. HC, *P*<0.01 vs. HT3, *P*<0.01 vs. HT3+10).

### ED1 positive cells, Myeloperoxidase activity and Collagen levels

**Figure 8:**
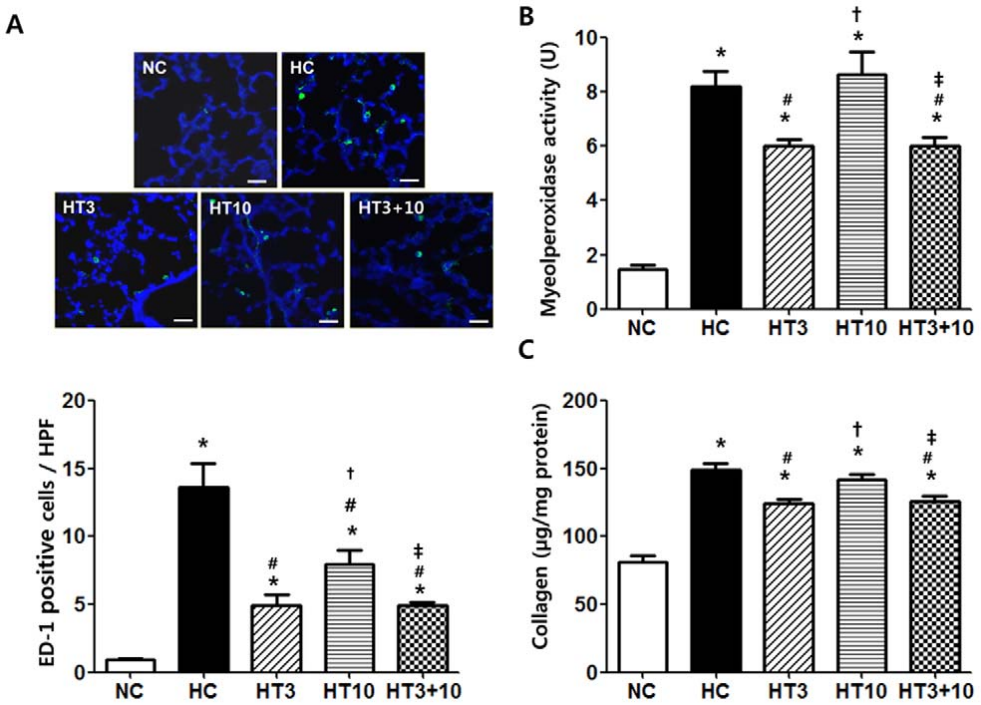
Inflammation with fibrosis in the hyperoxic lung injury after MSCs transplantation. ED1 positive cells indicative of alveolar macrophage were labeled with FITC (*green*) and the nuclei were labeled with DAPI (*blue*) (Scale bar; 25 µm) (*at top*) and number of observed ED1 positive cells per high power field (*below*) (A), myeloperoxidase activity (B), and collagen levels (C) of the rat P21 lung tissues. NC, Normoxia control group; HC, hyperoxia control group; HT3, hyperoxia with stem cell transplantation group at P3; HT10, hyperoxia with stem cell treatment group at P10; HT3+10, hyperoxia with stem cell treatment group at P3 and P10. Data; mean±SEM. ^*^ *P*<0.05 compared to NC, ^#^ *P*<0.05 compared to HC,^†^ *P*<0.05 compared to HT3, ^‡^ *P*<0.05 compared to HT10.

The ED1 positive alveolar macrophages were significantly higher in HC (13.6±1.8, *P*<0.001) than in NC (1.0±0.1). This hyperoxia- induced increase in ED1 positive cells was significantly attenuated with MSCs transplantation, and this attenuation was more profound in HT3 (4.9±0.8, *P*<0.001 vs. HC) and HT3+10 (4.9±0.2, *P*<0.001 vs. HC) than in HT10 (7.9±1.1, *P*<0.01 vs. HC, *P*<0.05 vs. HT3, *P*<0.05 vs. HT3+10) (Fig. 8A).

The MPO activity in HC (8.2±0.5 U, *P*<0.001) was significantly higher than in NC (1.5±0.2 U). The hyperoxia-induced increase in MPO activity was significantly attenuated in both HT3 (6.0±0.2 U, *P*<0.001 vs. HC) and HT3+10 (6.0±0.3 U, *P*<0.001 vs. HC), but not in HT10 (8.6±0.8 U, *P*>0.05 vs. HC, *P*<0.01 vs. HT3, *P*<0.01 vs. HT3+10) (Fig. 8B).

The lung collagen levels at P21 were significantly higher in HC (149±5 µg/mg protein, *P*<0.001) than in NC (81±5 µg/mg protein). This hyperoxia-induced increase in the lung collagen levels was significantly attenuated in both HT3 (124±3 µg/mg protein, *P*<0.01 vs. HC) and HT3+10 (126±4 µg/mg protein, *P*<0.01 vs. HC), but not in HT10 (142±4 µg/mg protein, *P*>0.05 vs. HC, *P*<0.05 vs. HT3, *P*<0.05 vs. HT3+10) (Fig. 8C).

### HGF and VEGF

**Figure 9:**
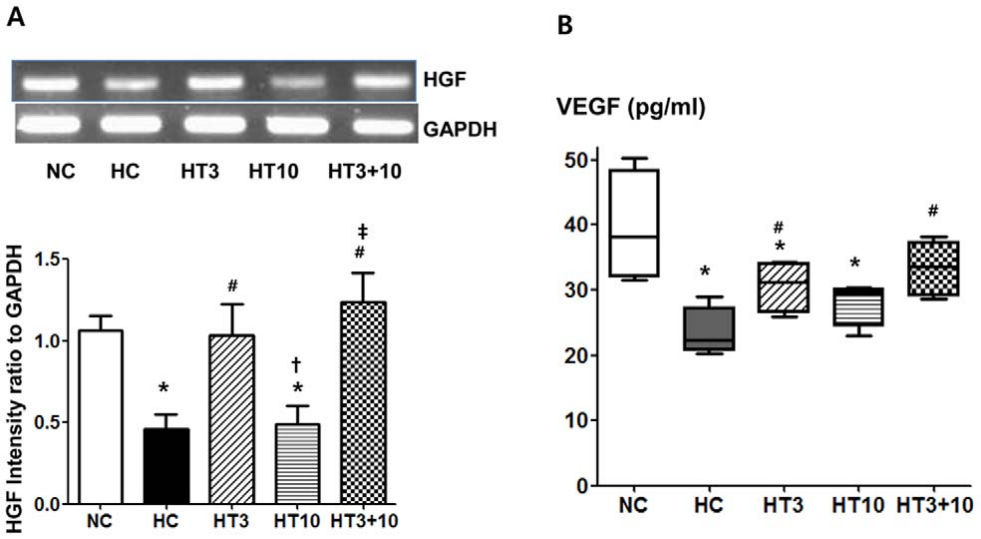
VEGF and HGF in the hyperoxic lung injury after MSCs transplantation. Representative RT-PCR blots (*at top*) and densitometric histograms (*below*) for HGF (A) and VEGF measured with ELISA (B) in the P21 rat lungs. NC, Normoxia control group; HC, hyperoxia control group; HT3, hyperoxia with stem cell transplantation group at P3; HT10, hyperoxia with stem cell treatment group at P10; HT3+10, hyperoxia with stem cell treatment group at P3 and P10. Data; mean±SEM. ^*^ *P*<0.05 compared to NC, ^#^ *P*<0.05 compared to HC,^†^ *P*<0.05 compared to HT3, ^‡^ *P*<0.05 compared to HT10.

The HGF in the rat lung measured by RT-PCR at P21 were significantly lower in HC (*P*<0.05) than in NC. The hyperoxia-induced decrease in lung HGF was significantly up-regulated in both HT3 (*P*<0.05 vs. HC) and HT3+10 (*P*<0.01 vs. HC), but not in HT10 (*P*>0.05 vs. HC, *P*<0.05 vs. HT3, *P*<0.01 vs. HT3+10) (Fig. 9A). The VEGF levels in the rat lungs measured by ELISA at P21 were significantly lower in HC (23.5±1.9 pg/ml, *P*<0.001) than in NC (39.5±4.3 pg/ml). This hyperoxia-induced decrease in the lung VEGF level was attenuated in both HT3 (30.6±2.0 pg/ml, *P*<0.05 vs. HC) and HT10 (33.4±1.6 pg/ml, *P*<0.01 vs. HC), but not in HT10 (28.1±1.7 pg/ml, *P*>0.05 vs. HC) (Fig. 9B).

## Discussion

In the present study, prolonged exposure of newborn rat pups to hyperoxia for 3 weeks increased mortality, retarded growth, and developed lung injuries similar to those seen in the premature human infants with BPD [23], [24], exhibiting decreased alveolarization as evidenced by increased MLI and alveolar volume [25], and significantly increased TUNEL positive cells [26]. Previously, we have shown that the neonatal hyperoxic lung injuries induced by ≥90% oxygen for 2 weeks were significantly attenuated with intratracheal human UCB-derived MSCs transplantation at P5 [7], and these beneficial effects were dose dependent [8]. In the present study, hyperoxic exposure was extended to 3 weeks to guarantee the comparable time span after MSCs transplantation at P10, and the oxygen concentration during the third week was reduced from 90% to 60% because of the concern about increased mortality due to prolonged high oxygen exposure. Only MSCs given at P3 but not at P10 showed protective effects against the hyperoxia-induced lung injuries, and no synergistic effects were observed with combined early+late at P3+10 MSCs transplantation in this study. Overall, these findings suggest that the therapeutic efficacy of human UCB-derived MSCs transplantation is time-dependent, showing protection only during the early but not late phase of inflammation.

In time course experiments of the present study, lung inflammatory cytokine levels such as TNF-α, IL-1α, IL-1β and IL-6 in HC became significantly higher after P5∼7 compared to NC. Intratracheal MSCs given at P3 or P5, but not at P10 protected against neonatal hyperoxic lung injuries in our previous [7], [8] and present studies. These findings suggest that increased secretion of proinflammatory cytokines and the ensuing full blown host inflammatory milieu might be a strong inhibitor of MSCs protection against neonatal hyperoxic lung injury, and thus the therapeutic time window of stem cell transplantation in BPD might be limited only to the early phase of inflammation.

In the present study, the number of PKH26 positive cells at P21, indicative of donor cell localization in the lung tissue, was significantly higher in HT10 and HT3+10 than in HT3. These findings suggest that substantial donor cell losses occur early after intratracheal MSCs transplantation [27]. However, significant protective effects against neonatal hyperoxic lung injuries were observed in both HT3 and HT3+10, but not in HT10. Similar results of beneficial effects of MSCs despite low engraftment have also been reported in other disease models such as acute renal failure [28], and cardiac injury [29]. In our previous study [7], [8], we also observed only very few donor cells differentiate into type II pneumocytes, Overall, these findings suggest that even a small number of MSCs survived could mediate their therapeutic effects against neonatal hyperoxic lung injuries primarily by secreting key bioactive mediators rather than by direct tissue repair [30], and early timing of MSCs transplantation is critical for their best protective effects. Moreover, although donor cells rapidly faded away after transplantation, the favorable effects of MSCs were persistent up to P21. These findings suggest that the paracrine effects initially induced by stem cells might play a pivotal role in tissue repair, and are sustained later by the intact host tissue protected by MSCs transplantation [31]. However, we cannot exclude the possibility that rejection might have occurred in our study because transplant was performed in a xenograft model; therefore, further studies are needed to clarify this.

Oxidative stress to the immature lung is a well-known risk factor for the development of BPD [32]. NADPH oxidase is a multi-component enzyme complex responsible for production of superoxide anion (O_2_^−^), which generates other reactive oxygen species such as hydrogen peroxide, hydroxyl radical, and hypochlorous acid [20]. Upon its activation, the cytoplasmic subunits p47*^phox^*, p67*^phox^*, p40*^phox^* and Rac translocate to membrane bound cytochrome [33]. The membrane translocation of the p47*^phox^* can thus be served as an *in vivo* indicator of NADPH oxidase activation in the lung tissue. In our previous study [8], we have observed the dose-dependent anti-oxidative effects of intratracheal MSCs transplantation. In the present study, increased expression of the p47*^phox^* protein was observed both in the cytosolic and membrane fraction in HC. Although significant attenuation of cytosolic expression of the p47*^phox^* was observed after MSCs transplantation, a decrease in membrane translocation of the p47*^phox^* was observed in both HT3 and HT3+10, but not in HT10. These findings suggest that MSCs transplantation at the early rather than late phase of inflammation will be the optimal timing for their best anti-oxidative effects.

In our previous studies [7], [8], we have shown that inflammatory responses mediated by neutrophils [4] and proinflammatory cytokines [5] play a pivotal role in the development of BPD [6], and that the protective effects of MSCs therapy against hyperoxia-induced lung injuries are mediated primarily by their anti-inflammatory effects rather than by their regenerating capacity. In the present study, hyperoxia-induced increase in ED1 positive alveolar macrophages, lung myeloperoxidase activity and cytokines such as IL-1α, IL-1β, IL-6, TNF-α measured by ELISA, other inflammatory markers such as TIMP-1, CXCL7, RANTES, L-selectin and sICAM-1 measured by protein array were consistently attenuated by MSCs administration at both early (HT3) and combined early+ late (HT3+10), but not at the late (HT10) phase of inflammation. These findings suggest a full-blown host inflammatory micro-environment might hinder the anti-inflammatory effects of the transplanted MSCs. Further studies will be necessary to elucidate the inhibitory mechanism of endogenous inflammatory factors on transplanted stem cells function.

In our previous studies [7], [8], we have shown that the anti-fibrotic effect of transplanted MSCs was associated and probably mediated by their down-modulation of hyperoxia-induced pulmonary inflammatory responses. Significant attenuation of hyperoxia-induced increase in both fibrogenic cytokines such as TGF-β and TIMP-1 [34] along with other inflammatory cytokines, and collagen levels in both HT3 and HT3+10, but not in HT10 support the assumption that the early timing of MSCs transplantation is critical for their best anti-inflammatory and the ensuing anti-fibrotic effects.

In the present study, hyperoxia-induced decrease in growth factors such as VEGF and HGF was significantly up-regulated in both HT3 and HT3+10, but not in HT10 despite higher donor cell localization in the lung tissue at P21 in HT10 than in HT3. These findings suggest that the protective effects of MSCs transplantation such as promotion of angiogenesis, anti-apoptotic effects and reduced inflammation are strongly associated or probably mediated by enhanced secretion of these growth factors [35]. Moreover, as the full blown host inflammatory milieu might hinder the secretion of these growth factors by MSCs [36] the timing of MSCs transplantation early in the inflammation is essential for enhanced expression of these growth factors. Further studies will be necessary to clarify this.

In summary, intratracheal transplantation of human UCB derived MSCs time-dependently attenuated hyperoxia-induced lung pathology such as decreased alveolarization and increased apoptotic cells, oxidative stress, inflammatory responses and the ensuing fibrosis, and up-regulated growth factors such as HGF and VEGF, showing significant protection only in the early at P3 but not in the late at P10 phase of inflammation. There were no synergies with combined early+late MSCs transplantation. These findings are expected to have important implications for future clinical translation to determine the optimal timing of MSCs transplantation for the currently untreatable neonatal hyperoxic lung disease i.e., BPD in premature infants.
